# Complementarity of Selective Culture and qPCR for Colistin Resistance Screening in Fresh and Frozen Pig Cecum Samples

**DOI:** 10.3389/fmicb.2020.572712

**Published:** 2020-11-09

**Authors:** Pedro Miguela-Villoldo, Miguel A. Moreno, Marta Hernández, David Rodríguez-Lázaro, Alejandro Gallardo, Carmen Borge, Alberto Quesada, Lucas Domínguez, María Ugarte-Ruiz

**Affiliations:** ^1^VISAVET Health Surveillance Centre, Universidad Complutense, Madrid, Spain; ^2^Departamento de Sanidad Animal, Facultad de Veterinaria, Universidad Complutense, Madrid, Spain; ^3^Laboratorio de Biología Molecular y Microbiología, Instituto Tecnológico Agrario de Castilla y León, Valladolid, Spain; ^4^Área de Microbiología, Departamento de Biotecnología y Ciencia de los Alimentos, Facultad de Ciencias, Universidad de Burgos, Burgos, Spain; ^5^Departamento de Bioquímica, Biología Molecular y Genética, Facultad de Veterinaria, Universidad de Extremadura, Cáceres, Spain; ^6^Departamento de Sanidad Animal, Facultad de Veterinaria, Universidad de Córdoba, Córdoba, Spain; ^7^INBIO G+C, Universidad de Extremadura, Cáceres, Spain

**Keywords:** freeze-thaw process, caecal samples, *mcr-1*, *mcr-2*, swine, antimicrobial resistance

## Abstract

Retrospective studies involving the screening of frozen stored collections of samples are commonplace when a new threat emerges, but it has been demonstrated that the freeze-thaw process can affect bacterial viability. The study of colistin-resistant bacteria in human and animal samples is an example of this issue. In this study, we compared culture-based and PCR-based methods for analyzing relative occurrence and diversity of colistin-resistant bacteria in caecal samples to determine the most appropriate method for frozen samples. Thus, 272 samples from the caecal contents of healthy pigs were tested before and after a 6-month freezing period. A selective medium was used when traditional isolation of colistin-resistant bacteria was tested, while a real-time SYBR^®^ Green I PCR assay was applied for *mcr-1* quantification. The number of samples with colistin-resistant isolates was higher in fresh samples (247/272) than in frozen ones (67/272) and showed a higher diversity of colistin-resistant genera. PCR identification of *mcr* colistin resistance genes evidenced that *mcr-1* was the most prevalent *mcr* gene and *mcr-2* was detected for the first time in pigs from Spanish animal production. The number of samples with *mcr-1*-carrying bacteria after a freezing period decreased, while real-time quantitation of the *mcr-1* gene showed similar values in frozen and fresh samples. Therefore, when frozen cecal samples need to be analyzed, molecular detection of DNA could be the best option to provide a highly representative frame of the initial population present in the sample, and culture-based methods might be a useful complement to study colistin resistance levels.

## Introduction

Retrospective studies involving the screening of stored frozen collections of isolates or, less frequently, biological samples are commonplace when a new microbiological threat emerges, and their aim is to test prior occurrences and features of the agent. One ongoing example is colistin-resistant bacteria in humans and animals. These isolates and samples usually belong to collections obtained in the past that have been kept frozen during a period of time. Temperature and time of storage are the most critical factors when a microbiological study is carried out with frozen material. In addition, the freeze-thaw process could be an important issue for bacterial recovery since it could dramatically affect bacterial survival because of the physical changes in the frozen material, assuming that at least a 74% decrease in cell viability may occur (Phalakornkule et al., 2017). This is of paramount importance when the aim is testing bacterial diversity of frozen samples, as previous studies have demonstrated that the freeze-thaw process can decrease bacterial diversity in the samples (Phalakornkule et al., 2017; Dorsaz et al., 2020). Therefore, the use of traditional methods, such as solid or liquid culture, could be challenging and may not provide representative data on the amount and diversity of bacteria in these samples prior to being frozen. On the other hand, DNA remains more stable after the freeze-thaw process as shown in different microbiome studies using Next Generation Sequencing (Bassis et al., 2017; Dorsaz et al., 2020). Similarly, the bacterial composition of stools was not dramatically affected after a freezing process using molecular methods, although this has been also associated with freezing conditions (Shao et al., 2012).

There are not many published studies that evaluate the effect of the freeze-thaw process comparing culture-based methods and molecular techniques as real-time PCR. Our work aims to evaluate how this process could affect the detection of colistin-resistant *Enterobacteriaceae* in frozen samples. Since the first description of a plasmid-mediated colistin resistance determinant in 2015 (*mcr-1*), up to ten *mcr*-related genes have been described. However, *mcr-1* still remains as the most common colistin resistance determinant carried by plasmids since it is present worldwide, especially in *Escherichia coli* of human and animal origin (Khedher et al., 2020; Wang et al., 2020); therefore, in this study we took *mcr-1* as an indicator of acquired colistin resistance. Many retrospective studies screened colistin resistance (both bacteria and genes) over bacterial strain collections (Carattoli et al., 2017; Garcia et al., 2018; Migura-Garcia et al., 2019), but they were rarely performed on biological samples (Miguela-Villoldo et al., 2019).

To provide more accurate insights on colistin resistance emergence and evolution, an analysis of samples from past years is required. For this reason, we tested and compared two different protocols, commonly used in routine bacterial screening to detect antimicrobial-resistant bacteria (Alba et al., 2018): selective culture (using a specific method to isolate colistin-resistant *Enterobaceriaceae*) and quantitative real-time PCR (targeting the *mcr-1* gene), in a collection of cecal samples from pigs. We aimed to determine the most appropriate method for testing these samples after the freezing period. In addition, we assessed the effect of the freeze-thaw process on the bacterial diversity of colistin-resistant bacteria in fresh and frozen samples using a selective culture medium.

## Materials and Methods

### Sampling

A total of 272 isolates from cecal contents of healthy pigs were sampled at slaughterhouses in different Spanish regions from September to December 2018. Feces were taken from the cecum, transported on ice (+4°C) and analyzed within 24 h by culture-based and molecular methods. Then, samples were diluted (1:10) in buffered peptone water and stored with glycerol at −40°C. Each sample was kept frozen for 6 months and analyzed again by the same methods.

### Culture-Based Methods

Samples were analyzed as described in Miguela-Villoldo et al. (2019) using ChromID Colistin R agar (ColR) (bioMérieux, France). Briefly, ColR plates were inoculated with 50 μL of a 500-fold BHI diluted sample with colistin (10 μg disk in BHI broth) and were incubated at 37^*o*^C for 24 h. Three different-colored morphologies (pink-burgundy, green and white-colorless) could grow in ColR plates according to manufacturers’ specifications. After incubation, one colony per morphology obtained in the ColR plate was subcultured in blood agar at 37^*o*^C for 24 h. The isolates obtained were identified by mass spectrometry using a Bruker Daltonics UltrafleXtrem MALDI TOF/TOF instrument (Bruker Daltonics, Bremen, Germany). Poorly identified isolates (score <2.3) were further analyzed by using the API^®^ 20-E *Enterobacteriaceae* identification kit (bioMérieux) and the specific PCR (Cabal et al., 2013) for pink-burgundy colonies to confirm *E. coli* isolates (associated to this morphology). After identification, molecular characterization of the isolates was carried out by a conventional PCR to analyze the presence of colistin resistance *mcr* genes (*mcr-1* to 5) (Rebelo et al., 2018). As described in literature, *mcr-6* seems to be an endogenous gene from *Moraxella* spp., *mcr-7* was only described in *Klebsiella* spp., *mcr-8* was identified in *Klebsiella pneumoniae* and *Raoutella ornithinolitica* and *mcr-9* and *mcr-10* do not seem to confer resistance to colistin (Khedher et al., 2020; Wang et al., 2020). Therefore, we focused on genes *mcr-1* to 5 since they have been described in different bacterial genera and have not been linked to a particular bacterial group.

In this study, we differentiate two types of isolates depending on the nature of their resistance to colistin: the ACRB group that includes bacterial genera naturally sensitive to colistin that have acquired resistance mechanisms, and NCRB which is composed of those genera that have intrinsic resistance to colistin (Olaitan et al., 2014; Lepelletier et al., 2018).

### Colistin MIC Determination From *mcr*-Carrying Bacteria

Colistin susceptibility testing was performed by the two-fold broth microdilution reference method according to ISO 20776–1:2019 (ISO 20776-1:2019, 2019) using Sensititre EUVSEC plates (Trek Diagnostic Systems, United States) having a colistin range from 1 to 16 mg/L. Colistin resistant isolates where those having a minimal inhibitory concentrations (MIC) higher than 2 mg/mL (Spellerberg and Fedor, 2003).

### DNA Extraction and Quantitative PCR

Direct DNA extraction from pig cecal samples was carried out using a commercial kit (FASTI001-1 FavorPrep Stool DNA Isolation Mini Kit, Favorgen-Europe, Vienna) and was coupled to a specific SYBR^®^ Green I (Thermo Fisher Scientific, Vilnius) real-time PCR assay for quantitative detection of the *mcr-1* gene (qPCR) described previously by Li et al. (2017). PCRs were run on a thermal cycler CFX 96 (Bio-Rad). The specificity of the primers has been confirmed by melting curve analysis. Four μL of each DNA elute were run in triplicate. Samples were considered positive when quantitative values were higher than 1.00 × 10^2^ per reaction (equivalent to 1.58 × 10^5^ copies/100 mg cecal content).

Aiming to test the qPCR protocol, we conduct a recovery assay using spiked samples. We used a pool of 10 g of feces sampled from pigs that had never been treated with antimicrobials, which were confirmed as negative to *mcr-1* genes by qPCR. The sample was diluted 1:10 in saline water (0.085% NaCl) to a 100 mL final volume and then divided into four aliquots of 20 mL (named A1, A2, A3, and A4). Three of them were spiked using a *mcr-1* positive strain of *E. coli* at three different concentrations (A1: 10^6^ CFU/mL; A2: 10^5^ CFU/mL; A3: 10^4^ CFU/mL), calculated from a 0.5 McFarland suspension (plate count of 5.3 × 10^7^ CFU/mL), being the last (A4), used as negative control. Direct DNA extraction from all the samples was coupled with the *mcr-1* specific real-time PCR assay for quantitative detection of the *mcr-1* gene included in our protocol.

In addition, another quantitative PCR was performed to detect and quantify the amount of *E. coli* present in the sample using a published PCR that used as a target *uidA* gene (Cabal et al., 2013).

### Statistical Analysis

The Shannon-Weaver index (H′) was calculated using results obtained after traditional culture to estimate genera diversity per sample (Spellerberg and Fedor, 2003):

$$H^{\prime }=-\sum_{i=1}^{s}p_{i}\,l\,n\,p_{i}$$

Where H′ is the biodiversity index, *i* is the genera and *p*_*i*_ = n_*i*_/N: where *n*_*i*_ is the total number of organisms of a particular genus and *N* is the total number of organisms of all genera. We used 95% confidence intervals (95% CI) to compare both fresh and frozen sample diversity indexes.

Statistical methods were used to analyze the data obtained by qPCR and the culture method; Fisher’s exact test was applied to compare the proportion of samples where *mcr-1*-carrying bacteria were obtained using a traditional culture method. In this case, at least one positive *mcr-1* isolate in the sample was needed to consider it positive, both in fresh and frozen samples. It was assumed that the freeze-thaw process had a significant effect on our results if *p*-value was less than 0.05. T-Test for two related samples was applied to analyze qPCR results. Data was previously normalized transforming it into Log_10_. A difference was considered significant when *p*-value was less than 0.05.

## Results

### Culture-Based Methods

#### Detection of Colistin-Resistant Bacteria

Colistin-resistant isolates were detected in 91% of fresh samples (247/272) and 24% of frozen-thawed samples (67/272) (*p*-value < 0.001). ACRB isolates were detected in 222/272 of fresh samples, (82%) and 60/272 of frozen-thawed samples (22%) (*p*-value < 0.001), whereas NCRB isolates were obtained from 140/272 of fresh samples (51%) and 12/272 of frozen-thawed samples (4%) (*p*-value < 0.001).

Regarding fresh samples, ACRB isolated genera were *Aeromonas*, *Citrobacter*, *Enterobacter*, *Escherichia, Klebsiella* and *Raoutella*. On the other hand, NCRB genera were *Morganella*, *Myroides*, *Proteus*, *Providencia*, and *Vibrio.* The diversity of genera found in fresh samples was estimated using Shannon-Weaver Index, obtaining a value of 1.496 (95% CI = [1.393, 1.599]). In these samples, *Escherichia* spp. was the most frequent genus identified (50% of fresh samples with bacterial growth had at least one *Escherichia* spp. isolate) (Table 1).

**TABLE 1:** Bacterial genera recovered by the culture method from fresh and frozen samples.

	Fresh Samples (*n* = 272)	Frozen Samples (*n* = 272)
Bacteria genera	*N*	*N*
*ARCB group*		
*Escherichia* spp.	212	56
*Klebsiella* spp.	20	2
*Citrobacter* spp.	9	1
*Raoutella* spp.	7	2
*Enterobacter* spp.	6	2
*Aeromonas* spp.	3	0
*NCRB*		
*Providencia* spp.	84	7
*Proteus* spp.	61	7
*Morganella* spp.	17	1
*Myroides* spp.	1	0
*Vibrio* spp.	1	0

*Up to three different genera could be grown in the same sample, thus, the table represents the number of samples where each genus has been detected. N: number of samples with bacterial growth. n: number of studied samples.*

ACRB obtained in frozen samples included genera such us *Citrobacter* spp., *Enterobacter* spp*., Escherichia* spp., *Klebsiella* spp., and *Raoutella* spp. Meanwhile, NCRB were represented by *Morganella* spp., *Proteus* spp. and *Providencia* spp. Shannon-Weaver Index, taking into account the genera observed, obtaining a value of 1.064 (95%CI = [0.782, 1.34]). In this case, *Escherichia* spp. was also the most frequent bacterial genus (72% of frozen samples with bacterial growth had at least one *Escherichia* spp. isolate) (Table 1).

The minimum overlapping between confidence intervals of Shannon-Weaver indexes from both fresh samples ([1.393, 1.599]) and frozen samples ([0.782, 1.346]) showed that diversity decreased in samples after the freezing process.

Interestingly, our results showed that the freeze-thaw process affected ACRB and NCRB differently. While NCRB experimented a 92% decrease in the number of isolates obtained, ACRB only decreased by 77%. Finally, the freeze-thaw process decreased viability to 84% (from 486 to 78 in fresh and frozen samples, respectively).

#### Molecular Detection of *mcr* Genes in Isolates Recovered by the Culture Method

From a total of 272 cecal samples, *mcr* genes were identified in 77% (209/272) and 21% (57/272) of fresh and frozen ones, respectively. Similarly, while the *mcr-1* gene was detected in 51.4% (250/486) of the isolates obtained from fresh samples, *mcr-4* was identified in four, one of them in co-occurrence with *mcr-1* (*E. coli*), and *mcr-2* in one *E. coli* isolate. The proportion of *mcr*-carrying isolates in the ACRB group was 86.4 and 92.1% for fresh and frozen samples, respectively. Finally, no *mcr* gene was found in 40 putative ACRB isolates; colistin resistance in those cases may thus be due to other mechanism(s). The remaining 192 isolates lacking *mcr-1-5* genes were NCRB.

However, *mcr-1* was detected in 73.07% (57/78) of the isolates from frozen samples (including all genera detected), while *mcr-4* was identified in one *E. coli*, all of them being ACRB. The remaining 21 isolates were PCR-negative for the *mcr* genes tested (6 ACRB and 15 NCRB) (Table 2).

**TABLE 2:** Molecular detection of *mcr* genes (*mcr-1* to *5*) by conventional PCR.

	Fresh samples (272 samples, 486 isolates)						Frozen samples (272 samples, 78 isolates)					
	*mcr-1*	*mcr-2*	*mcr-4*	*mcr-1*/*mcr-4*	No *mcr*	Total	*mcr-1*	*mcr-2*	*mcr-4*	*mcr-1*/*mcr-4*	No *mcr*	Total
ACRB group												
*Escherichia* spp.	236	1	1	1	10	249	54	0	1	0	1	56
*Klebsiella* spp.	7	0	1	0	12	20	2	0	0	0	0	2
*Citrobacter* spp.	5	0	1	0	3	9	1	0	0	0	0	1
*Raoutella* spp.	1	0	0	0	6	7	0	0	0	0	2	2
*Enterobacter* spp.	0	0	0	0	6	6	0	0	0	0	2	2
*Aeromonas* spp.	0	0	0	0	3	3	NA	NA	NA	NA	NA	0
Total	249	1	3	1	40	294	57	0	1	0	5	63
*ACRB mcr*%	85.70	0.34	1.02	0.34	13.60		90.50		1.59		7.94	
*mcr-carrying ACRB proportion*						86.40%						92.10%
NCRB group												
*Providencia* spp.	0	0	0	0	105	105	0	0	0	0	7	7
*Proteus* spp.	0	0	0	0	68	68	0	0	0	0	7	7
*Morganella* spp.	0	0	0	0	17	17	0	0	0	0	1	1
*Myroides* spp.	0	0	0	0	1	1	NA	NA	NA	NA	NA	0
*Vibrio* spp.	0	0	0	0	1	1	NA	NA	NA	NA	NA	0
Total	0	0	0	0	192	192	0	0	0	0	15	15
NCRB *mcr*%					100						100	
Total *mcr%*	51.2	0.21	0.62	0.21	47.74		73.08		1.28		25.64	

*All isolates obtained from fresh and frozen samples are represented in this table, independently of the total number of samples. NA, “Not Applicable” values correspond to genera that have not been found in the sample, so no analysis could be applied. ACRB, Acquired colistin-resistance bacteria. NCRB, Natural colistin-resistance bacteria.*

#### Colistin Susceptibility of *mcr*-Carrying Isolates

Colistin MIC values were checked in the 312 *mcr*-carrying isolates from both fresh and frozen samples (Table 3). From a total of 254 *mcr*-carrying ACRB isolates obtained from fresh samples, 168 (66.2%) had a MIC value of 4 μg/mL, 79 isolates (31.0%) 8 μg/mL, two isolates 16 μg/mL and two more a MIC higher than 16 μg/mL. Three *mcr-1* isolates showed a MIC of 2 μg/mL.

**TABLE 3:** Distribution of colistin MIC values of *mcr*-carrying isolates obtained from both fresh and frozen samples.

		Number of isolates						

		Dilution test range: 1 – 16 μg/ml colistin MICs (μg/ml)						
	*mcr* pattern	≤1	2	4	8	16	>16	Total
Fresh samples	*mcr-1*	0	3	168	75	2	1	248
	*mcr-2*	0	0	0	1	0	0	1
	*mcr-4*	0	0	0	2	0	1	3
	*mcr-1/mcr-4*	0	0	0	1	0	0	1
	*Total*	0	3	168	79	2	2	254
Frozen samples	*mcr-1*	0	0	28	28	0	1	57
	*mcr-2*	0	0	0	0	0	0	0
	*mcr-4*	0	0	0	1	0	0	1
	*mcr-1/mcr-4*	0	0	0	0	0	0	0
	*Total*	0	0	28	29	0	1	58

Regarding frozen samples, from a total of 58 *mcr*-carrying ACRB isolates, 28 (48.3%) showed a MIC of 4 μg/mL, 29 (50%) 8 μg/mL and one isolate more than 16 μg/mL.

#### Detection of *mcr-1* Isolates in Cecal Samples

As summary, from the 272 fresh samples tested, 208 (76%) showed a growth of *mcr*-1 positive bacteria, all of them being ACRB. On the other hand, 56 frozen samples (20%) showed growth of *mcr*-1 positive bacteria, all being ACRB as well. The total number of isolates testing positive to the *mcr*-1 gene, obtained in both fresh and frozen samples, can be seen in Table 2, taking into account that in each sample up to three different colony morphologies could be detected. Fisher’s exact test provided a *p*-value of 0.0001 comparing results of *mcr*-1 positive samples in fresh and frozen samples, the difference observed being statistically significant.

### Quantification of *mcr-1* by Real-Time PCR

Regarding the recovery assay, qPCR reaction was carried out with 91.27% efficiency, *r*^2^ value of 0.999 and 85°C melt temperature. Cycle thresholds (cts) of the standard curve were 18.08 (sd = 0.113), 21.77 (sd = 0.174), 25.52 (sd = 0.135), 29.37 (sd = 0.266), and 32.98 (sd = 0.532) for standard 1 to 5, respectively. The results of the *mcr-1* qPCR were as follows: 7.22 × 10^9^ copies/g, 8.08 × 10^8^ copies/g, 9.22 × 10^7^ copies/g and < 10^1^ copies/g from A1, A2, A3, and A4, respectively, and a relative accuracy of 100.01%, 99.97% and 100.02% for A1, A2, and A3, respectively. These results agreed to those expected, taking into account that this test corresponds to a total *mcr-1* quantification.

The results of the *uidA* quantitative real-Time PCR were as follows: 1.60 × 10^10^ *uidA* copies/g, 6.43 × 10^9^ *uidA* copies/g, 6.73 × 10^9^ *uidA* copies/g, 3.96 × 10^9^ *uidA* copies/g from A1, A2, A3, and A4, respectively.

**FIGURE 1:**
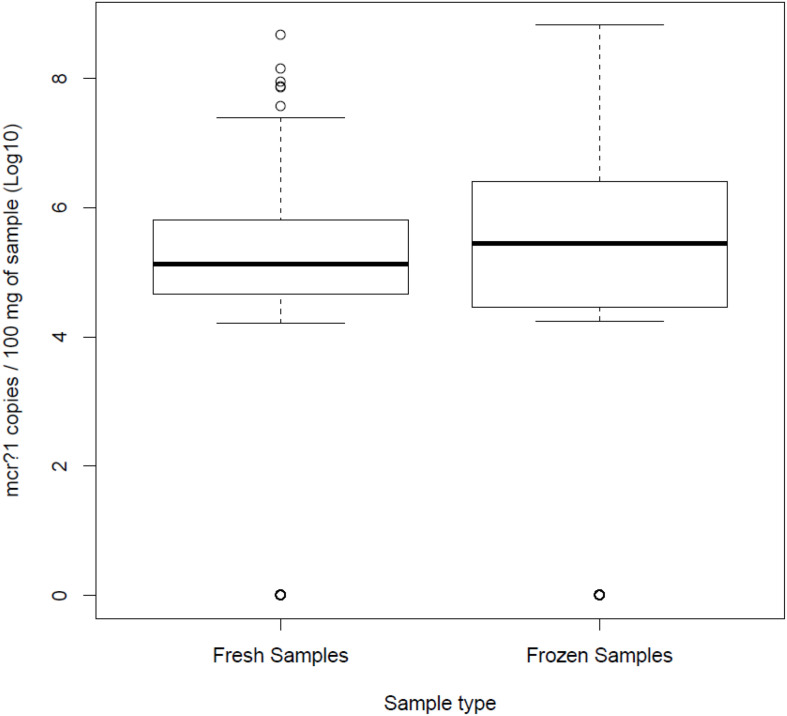
Quantitative real-time PCR results for quantitation of the *mcr-1* gene in both fresh and frozen samples.

From a total of 272 samples per group, the *mcr-1* gene was quantified in 128 fresh (47%) and 142 frozen samples (52%), with mean values of 4.75 Log_10_ copies/100 mg feces and 4.66 Log_10_ copies/100 mg feces, respectively (Figure 1), differences that were not statistically significant (*p* = 0.508, *T*-test).

### Culture and PCR in Frozen Samples

From a total of 272 frozen samples analyzed, equivalent results were obtained from 176 samples (51 samples were positive, 125 were negative), whereas 96 showed discrepancies, mainly due to 91 samples that were quantified by qPCR but not detected by culture methods and only five samples were qPCR negative being positive by culture.

## Discussion

The freezing process could have a negative effect on the viability of bacteria present in cecal samples, which may affect retrospective studies based on the detection of bacteria using culture methods. In this study, we use freezing in glycerol to sample storage, a process involving physical and chemical changes that could affect their biological composition (Phalakornkule et al., 2017). Thawing-freezing is critical for bacterial viability and for decreasing their recoverability. The effect of this process must be considered in the study design, data analysis and interpretation of the results (Phalakornkule et al., 2017). In our study, fecal samples showed a higher diversity of colistin-resistant bacteria when tested prior to freezing, and a significant decrease was confirmed in both occurrence and diversity after freezing. Besides, the number of samples with colistin-resistant isolates was higher in fresh (247/272) than in frozen samples (67/272), according to the expected bacterial viability level after the freeze-thaw process previously described (Phalakornkule et al., 2017). Regarding both ACRB and NCRB groups, we noticed that the freeze-thaw process affected these two groups differently, since NCRB (192 in fresh samples vs. 15 in frozen samples) showed a higher decrease in the number of isolates obtained than ACRB (294 in fresh samples vs. 63 in frozen samples), although further analyses are needed to determine the cause. Our results showed that the freeze-thaw process drastically reduced bacterial viability, affecting some bacterial genera differently and altering the microbiological diversity of stored samples.

Regarding molecular detection of plasmid-mediated *mcr* genes, our results evidenced that *mcr-1* was the most prevalent colistin-resistance gene in our sample collection among the *mcr-1* to *mcr-5* group of determinants, in accordance with previous studies (Carattoli et al., 2017; Poirel et al., 2018; Migura-Garcia et al., 2020). We also identified *mcr-2* and *mcr-4* genes. To our knowledge, we report the first description of *mcr-2* in healthy pigs for food production in Spain and the second in Europe after its discovery in Belgium in 2016 (Xavier et al., 2016). This gene was identified in one *E. coli* (serotype O83:H42) belonging to ST-648 and showing 100% similarity to *mcr-2.1* described previously (Xavier et al., 2016). Most of these *mcr*-carrying isolates showed a colistin MIC value higher than 2, being 4 and 8 μg/mL the two most frequent MICs detected, as described in other studies (Xavier et al., 2016; Carattoli et al., 2017; Poirel et al., 2017; Lepelletier et al., 2018).

In this study, we compared both molecular and culture-based methods, focusing on those samples showing growth of ACRB carrying *mcr-1*, the main colistin-resistance determinant that has been quantified by SYBR^®^ Green qPCR. The amount of *mcr-1* gene detected was similar between frozen and fresh samples, suggesting that the freeze-thaw process did not significantly modify the availability of functional DNA for the qPCR, in line with other studies that compared PCR results before and during different freezing periods (Bassis et al., 2017; Dorsaz et al., 2020). However, PCR may overestimate the content in viable cells showing colistin resistance of cecal samples, since dead cells could also be detected (Dorsaz et al., 2020).

Regarding samples with *mcr-1* positive isolates, freezing significantly decreases bacterial viability, while *mcr-1* DNA remained stable as detected by qPCR (Figure 1). This suggests that the use of culture-based methods for testing frozen samples might underestimate the occurrence of plasmidic colistin-resistance determinants in cecal samples, as it could be observed when comparing qPCR data with culture data. However, these methods provided information about bacterial diversity of biological samples which could not be achieved by using qPCR. Therefore, although culture and molecular methods differ in properties to detect colistin resistance, and mainly the specificity to detect viable cells or the sensitivity to quantify functional DNA molecules, both methods are complementary techniques used to characterize biological samples depending on the objectives of the study; taking into account that, while bacterial viability decreases as a result of the freezing, the DNA concentration remained stable.

## Conclusion

Data obtained from selective culture showed that both bacterial viability and diversity of colistin-resistant genera were reduced after the freeze-thaw process, involving the loss of members of the bacterial population that was initially present in the sample. In contrast, *mcr-1* detection by qPCR directly on fresh or frozen samples produced similar results. Thus, detection of DNA by qPCR when frozen samples need to be analyzed would be the option of choice to provide a highly representative frame of the spectrum of resistance determinants initially present in the sample, and culture-based methods would be the best complement to detect the carriage of viable and colistin-resistant cells.

## Data Availability Statement

The raw data supporting the conclusions of this article will be made available by the authors, without undue reservation.

## Ethics Statement

Ethical review and approval was not required for the animal study because no animal was manipulated in this study, only samples of cecal content taken at slaughterhouse.

## Author Contributions

PM-V, MM, LD, and MU-R: conceptualization, investigation, resources, data curation, writing – original draft preparation, project administration, and funding acquisition. PM-V, MH, and DR-L: methodology. MH, DR-L, and AQ: software and validation. PM-V, MH, MM, DR-L, AQ, LD, and MU-R: formal analysis, writing – review and editing, visualization, and supervision. All authors contributed to the article and approved the submitted version.

## Conflict of Interest

The authors declare that the research was conducted in the absence of any commercial or financial relationships that could be construed as a potential conflict of interest.
